# Development and evaluation of a kidney health questionnaire and estimates of chronic kidney disease prevalence in the Cooperative Health Research in South Tyrol (CHRIS) study

**DOI:** 10.1007/s40620-024-02157-6

**Published:** 2024-11-27

**Authors:** Giulia Barbieri, Lucia Cazzoletti, Roberto Melotti, Essi Hantikainen, Rebecca Lundin, Laura Barin, Martin Gögele, Peter Riegler, Pietro Manuel Ferraro, Peter Paul Pramstaller, Giovanni Gambaro, Maria Elisabetta Zanolin, Cristian Pattaro

**Affiliations:** 1 Institute for Biomedicine, Eurac Research, Via Volta 21, 39100 Bolzano/Bozen, Italy; 2 Unit of Epidemiology and Medical Statistics, Department of Diagnostics and Public Health, Università Degli Studi Di Verona, Verona, Italy; 3 Independent Researcher, Bolzano/Bozen, Italy; 4 Section of Nephrology, Department of Medicine, Università Degli Studi Di Verona, Verona, Italy

**Keywords:** Cooperative Health Research in South Tyrol (CHRIS) study, Chronic kidney disease, Prevalence, Questionnaire, Awareness, CKD

## Abstract

**Background:**

Kidney diseases are a public health burden but are poorly investigated in the general population. In light of inadequate survey tools, we developed a novel questionnaire for use in population-based studies, to retrospectively assess kidney diseases.

**Methods:**

The questionnaire covered general kidney diseases, reduced kidney function, and renal surgeries. It was administered between 2011 and 2018 to 11,684 participants (median age = 45 years) of the Cooperative Health Research in South Tyrol (CHRIS) study. Fasting estimated glomerular filtration rate (eGFR) and urinary albumin-to-creatinine ratio (UACR) were measured. By factor analysis we contextualized the questionnaire content with respect to the biochemical measurements. We estimated overall and sex-stratified prevalence of kidney diseases, including possible CKD, calibrating them to the general target population via relative sampling weights.

**Results:**

Population-representative prevalence of glomerulonephritis, pyelonephritis, and congenital kidney diseases was 1.0%, 3.0%, and 0.2%, respectively, with corresponding odds ratios for females versus males of 1.4 (95% confidence interval: 1.0, 2.0), 8.7 (6.2, 12.3), and 0.7 (0.3, 1.6), respectively. Prevalence of kidney dysfunction (eGFR < 60 mL/min/1.73 m^2^ or UACR > 30 mg/g) was 8.59%, while prevalence of self-reported CKD was 0.69%, indicating 95.3% of lack of disease awareness, with a similar figure in people with diabetes or hypertension. Overall, 15.76% of the population was affected by a kidney disease of any kind.

**Conclusion:**

In the Val Venosta/Vinschgau alpine district, CKD prevalence aligned with Western European estimates. Kidney health questionnaire implementation in population studies is feasible and valuable to assess CKD awareness, which we found to be dramatically low.

**Graphical abstract:**

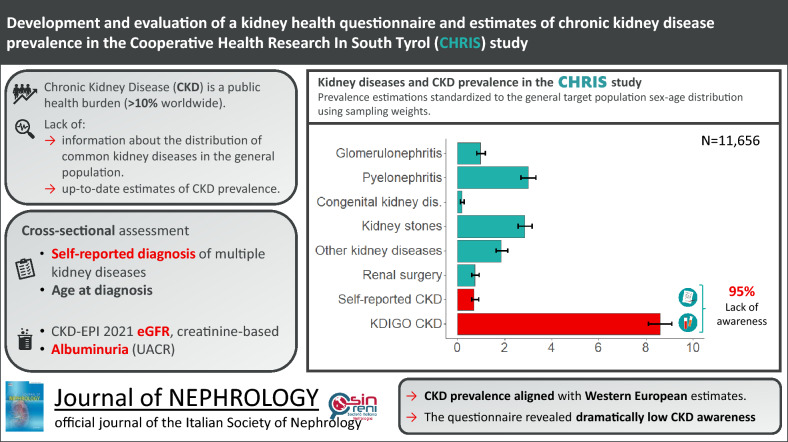

**Supplementary Information:**

The online version contains supplementary material available at 10.1007/s40620-024-02157-6.

## Introduction

Chronic kidney disease (CKD) is becoming one of the leading causes of death worldwide. It affects individual health and quality of life, significantly burdening healthcare systems. With a global prevalence above 10%, and significant geographic variations both between-[1] and within-continents [2], the distribution of CKD is influenced by environmental, behavioral, and genetic determinants, and public health policies [2, 3]. Hence, locally monitoring CKD prevalence is crucial in controlling this chronic disease.

In Italy, the latest estimates of CKD prevalence date back a decade. Based on estimated glomerular filtration rate (eGFR) and albuminuria measurements, CKD prevalence was estimated at 12.7% in 40 + year-old individuals [4] and 7.1% in those 35–79 years old [5]. These figures indicated an increase compared to the previous decade, with age-adjusted CKD prevalence at 5.7% for males and 6.2% for females [6]. The same study observed that less than 6% of the prevalent cases reported a diagnosis of any kidney disease. This gap between measured and self-reported CKD diagnosis aligns with the much lower standardized CKD prevalence (1.8%) estimated in the Lazio region (Italy) using a classification algorithm based on administrative data [7], reflecting the critical issue of CKD underdiagnosis [8, 9].

There are scant data on the prevalence of specific kidney diseases, such as glomerulonephritis [10, 11], and pyelonephritis. Except for measured blood and urinary markers, tools for surveying CKD and other kidney-related conditions in the general population remain limited. For this reason, we designed a questionnaire to identify broad categories of kidney diseases for application to general population studies. The questionnaire was implemented in a sizable central-European population study conducted in the Val Venosta/Vinschgau district (Italy), i.e., the Cooperative Health Research in South Tyrol (CHRIS) study.

The aim of this investigation was to analyze the novel kidney health questionnaire introduced in the CHRIS study, with attention to each reported condition, the internal relations between questionnaire items, and their relation with eGFR and albuminuria measured at the time of the interview. To characterize the kidney health context of the region, we additionally estimated population-standardized, overall and sex-stratified prevalence of any kind of kidney disease by combining questionnaire data with eGFR and albuminuria. Finally, we quantified kidney dysfunction awareness and underdiagnosis in the region.

## Methods

### Study design

Between 2011 and 2018, the CHRIS study enrolled 13,388 consenting adults living in the Vinschgau/Val Venosta community district (South Tyrol, Italy) [12]. From an administrative point of view, the study area comprises 13 municipalities grouped into an administrative entity known as the “Comunità Comprensoriale Val Venosta / Bezirksgemeinschaft Vinschgau”, formally constituted in 1962. The region is a typical Alpine environment, with a main valley and several side valleys inhabited by rural communities organized into little towns, small villages and scattered farms. The region is predominantly German-speaking (97%) with an Italian-speaking minority (3%). The resident population is relatively stable, with limited emigration, sharing a more homogeneous lifestyle compared to large urban settings [13, 14].

Following overnight fasting, participants underwent a blood draw, urine collection, blood pressure and anthropometric measurements, and clinical examinations. They filled out standardized questionnaires investigating demographics, medical history, and lifestyle. Barcodes of the drugs taken in the previous week were scanned and Anatomical Therapeutic Chemical (ATC) codes were obtained.

### The CHRIS kidney questionnaire

To identify different kidney diseases, we developed a dedicated questionnaire based on typical diagnoses and previous experience from a smaller study in the same district [15, 16]. The CHRIS kidney questionnaire, developed in German (the most commonly-spoken language in the district) and Italian, and translated into English, was administered in person by trained study assistants. The first year of the study was run as a pilot phase and included 1698 participants (57.5% females, median age 46.2 years), who were then excluded from our analysis. Afterwards, the questionnaire (Supplemental Figure S1) was re-evaluated and improved. The subsequent final version consists of a multiple-choice, retrospective questionnaire (Supplemental Table S1) with a nested structure divided into three sections. Section S1, on specific kidney diseases, started by screening whether a doctor ever told the participant they had a kidney disease of any kind (Q0) and, if yes, asked if they ever had a diagnosis of six specific diseases, namely: glomerulonephritis (Q1), pyelonephritis (Q2), renal artery disease (Q3), hereditary or congenital kidney disease (Q4), kidney stones (Q5), and any other kidney disease not mentioned before (Q6). Each disease had a sub-question on the age at diagnosis and a free-text field. For diseases affecting the glomeruli, we used “glomerulonephritis” as more specific terms would have been difficult to categorize, and because participants frequently reported it in a previous study [16]. Section S2 included questions on reduced kidney function (Q7, “*Has a doctor ever told you that you have a reduced kidney function or a renal failure?*”) as well as questions on dialysis (Q8) and transplants (Q9). Section S3 dealt with renal surgeries (Supplemental Table S1). We analyzed the results of all items except dialysis (Q8, 0 cases), transplantation (Q9, 0 cases), kidney donations (Q10, 0 cases), renal artery diseases (Q3, 5 cases), and surgeries (Q11, 2 cases), due to low or null case numbers.

### Biochemical markers of kidney health

Serum and urine creatinine were measured with the Jaffe method (Roche Modular PPE and Abbott Diagnostic Architect c16000 instruments) [17]. Urinary albumin was measured with immunonephelometry. Albumin values below the assay limit of detection were set to the limit. GFR was estimated with the race-free 2021 CKD-EPI equation [18]. Kidney damage was assessed as the urinary albumin-to-creatinine ratio (UACR). Changes in the measurement assays [17] were addressed via quantile normalization to the most recent assay [19]. UACR was log-transformed (logUACR) to mitigate skewness.

### Kidney disease definitions

The study design did not allow to confirm the persistence of abnormal eGFR and UACR after at least 3 months as prescribed by KDIGO guidelines [20]. Taking this into account, we defined “increased albuminuria” as UACR > 30 mg/g; “reduced eGFR” as eGFR < 60 mL/min/1.73 m^2^; “kidney dysfunction” as increased albuminuria or reduced eGFR; “self-reported CKD” (CKD_SR_) as a positive response to Q7 “*Has a doctor ever told you that you have a reduced kidney function or a renal failure?*”; “possible CKD” as either kidney dysfunction or CKD_SR_;[3] and “kidney disease of any kind” as possible CKD or any other self-reported kidney disease.

### Comorbidities

Hypertension was defined as: (i) an affirmative answer to “*Has a doctor ever said that you have high blood pressure or hypertension?*”; (ii) current blood pressure lowering therapy; or (iii) measured systolic or diastolic blood pressure of ≥ 140 or ≥ 90 mmHg, respectively. Diabetes mellitus was defined as: (i) an affirmative answer to “*Have you ever been diagnosed with diabetes by a doctor?*”; (ii) current diabetes medication; or (iii) measured HbA1c ≥ 6.5%. ATC codes for hypertension and diabetes mellitus definition are reported in Supplemental Table S2.

### Statistical analyses

We excluded 1698 participants filling in the first version of the kidney questionnaire, 6 without kidney questionnaire, and 28 with missing marker measurements, leaving 11,656 participants for statistical analyses (Supplemental Figure S2). All prevalence and proportion estimates were standardized to the sex and age structure of the target population via post-stratified relative sampling weights. Prevalence confidence intervals (CIs) were estimated using the Clopper-Pearson method [21]. For categorical variables with > 2 levels, multinomial proportion CIs were estimated by the Wald method [22].

For each kidney questionnaire item, sex differences were evaluated through age-adjusted logistic regression models with population sampling weights to account for variations in representativeness. We estimated pairwise tetrachoric correlation between all questionnaire items, conducting exploratory factor analysis to assess the correlation structure using only the questionnaire items. In a second exploratory factor analysis, we introduced increased albuminuria and reduced eGFR to explore the questionnaire’s ability to identify participants with reduced kidney function. The optimal number of factors was determined via scree plot examination. Sensitivity and specificity of each questionnaire item were assessed against reduced eGFR, increased albuminuria, and kidney dysfunction as gold standards. Regarding the question “*Has a doctor ever told you that you have a reduced kidney function or a renal failure?*” (Q7), we analyzed the distribution of eGFR and logUACR against responses (‘Yes’, ‘No’, and ‘I do not know’), through sex- and age-adjusted linear models, including Q7 responses as a categorical variable. Models were replicated using a six-category exposure variable combining Q7 responses with presence and absence of hypertension and/or diabetes mellitus.

### Software

All analyses were performed with the R software v4.1.1 (https://www.r-project.org/) using: the *tetrachoric* and *fa.poly* functions of the ‘psych’ package v2.2.5 for tetrachoric correlation and factor analyses, respectively (https://cran.r-project.org/web/packages/psych/index.html); the *confusionMatrix* function of the ‘caret’ package v6.0–93 for sensitivity and specificity analyses [23]; the *BinomCI* and *MultinomCI* functions of the ‘DescTools' package v0.99.48 for CI estimation of prevalence and proportions (https://cran.r-project.org/web/packages/DescTools/index.html); the ‘nephro’ package v1.3.0 for GFR estimation [15].

## Results

### Study sample characteristics

The 11,656 participants had a median age of 45.5 years (interquartile range, IQR = 30.9–57.3, range = 18–93.6),(Supplemental Figure S3) and 53.8% were females (Table 1). eGFR and UACR had median levels of 98.4 ml/min/1.73 m^2^ (IQR = 87.8–108.8) and 5.7 mg/g (IQR = 3.8–10.0), respectively, and were slightly negatively correlated (Supplemental Figure S4). Females had lower eGFR and higher UACR than males.

**Table 1:** Study sample characteristics

Characteristics	N (%) or median (IQR)		
	Overall	Males	Females
Sample size (%)	11,656 (100)	5384 (46.2)	6272 (53.8)
Age, years			
Median (IQR)	45.5 (30.9, 57.3)	46.1 (31.5, 58.1)	45 (30.4, 56.7)
Min–Max	18.0–93.6	18.0–93.5	18.0–93.6
Education, *N* (%)			
Primary school or no title	1225 (10.5)	475 (8.8)	750 (12.0)
Lower secondary school	1856 (15.9)	757 (14.1)	1099 (17.5)
Upper secondary school	2708 (23.2)	1010 (18.8)	1698 (27.1)
Vocational school	4813 (41.3)	2759 (51.2)	2054 (32.7)
University or higher	1054 (9.0)	383 (7.1)	671 (10.7)
eGFR, ml/min/1.73 m^2^			
Median (IQR)	98.4 (87.8, 108.8)	100.7 (89.9, 111.3)	96.2 (86.4, 106.6)
Min–Max	23.7–136.4	23.7–136.4	26.8–133.7
UACR, mg/g			
Median (IQR)	5.7 (3.8, 10.0)	4.4 (3.2,7.2)	7.2 (4.7,12.1)
Min–Max	1.3–2904.5	1.4–2904.5	1.3–2623.1
Diabetes mellitus, *N* (%)	359 (3.1)	168 (3.1)	191 (3.0)
Hypertension, *N* (%)	3623 (31.1)	1901 (35.3)	1722 (27.5)
At least one comorbidity (DM or HT)	3712 (31.8)	1932 (35.9)	1780 (28.4)

Abbreviations: *IQR* interquartile range; *eGFR* estimated glomerular filtration rate; *UACR* urinary albumin-to-creatinine ratio; *DM* diabetes mellitus; *HT* hypertension

### Analysis of the CHRIS kidney questionnaire

On the questionnaire, 743 (6.6%) participants reported diagnoses of one kidney disease and 183 (1.6%) two or more kidney diseases, with significant sex differences (Table 2). Pyelonephritis (3.0%), kidney stones (2.9%), and other unspecified kidney diseases (1.9%) were the most reported. Females reported less kidney stones (odds ratio, OR = 0.7, 95%CI 0.5–0.8) and renal surgeries (OR = 0.6, 95%CI 0.4–0.9), and more pyelonephritis (OR = 8.7, 95%CI 6.2–12.3) than males. Years since diagnosis showed disease-dependent distributions (Table 2; Supplemental Figure S5): the median time from diagnosis was 30 years for glomerulonephritis (IQR = 11–43), 29 for pyelonephritis (IQR = 15–39) and 26 for congenital kidney diseases (IQR = 19–32). Kidney stones (median = 14, IQR = 6–26), other kidney diseases (median = 10, IQR = 3–24), and reduced kidney function or renal failure (median = 10, IQR = 3–27) were diagnosed more recently.

**Table 2:** Overall and sex-stratified prevalence of self-reported kidney diseases calibrated to the general population distribution

Questionnaire item^a^	Absolute overall frequency No/Yes (D.K.)	Years from diagnosis: median (IQR)	Prevalence (95% confidence interval^b^)			OR^c^ (F vs M)
			Overall	Males	Females	(reference: M)
Q1: Glomerulonephritis	11,443/110 (103)	30 (11, 43)	1.0 (0.8, 1.2)	0.8 (0.6, 1.1)	1.2 (0.9, 1.5)	1.4 (1.0, 2.0)
Q2: Pyelonephritis	11,205/359 (92)	29 (15, 39)	3.0 (2.7, 3.3)	0.6 (0.4, 0.9)	5.4 (4.8, 6.0)	8.7 (6.2, 12.3)
Q4: Hereditary/congenital kidney disease	11,575/20 (61)	26 (19, 32)	0.2 (0.1, 0.3)	0.2 (0.1, 0.4)	0.1 (0.1, 0.3)	0.7 (0.3, 1.6)
Q5: Kidney stones	11,280/311 (65)	14 (6, 26)	2.9 (2.6, 3.2)	3.3 (2.9, 3.8)	2.4 (2.0, 2.8)	0.7 (0.5, 0.8)
Q6: Other kidney diseases	11,400/200 (56)	10 (3, 24)	1.9 (1.6, 2.1)	1.8 (1.4, 2.1)	2.0 (1.6, 2.4)	1.1 (0.8, 1.4)
Q7: Reduced kidney function	11,550/73 (33)	10 (3, 27)	0.7 (0.5, 0.9)	0.6 (0.5, 0.9)	0.7 (0.5, 1.0)	1.1 (0.7, 1.7)
Q12: Renal surgery	11,575/75 (6)	12 (4, 31)	0.7 (0.6, 0.9)	0.9 (0.7, 1.2)	0.6 (0.4, 0.8)	0.6 (0.4, 0.9)

Number of self-reported kidney diseases	Absolute overall frequency		Proportions (95% confidence interval^d^)			OR^e^ (F vs M)
			Overall	Males	Females	
None	10,730		91.7 (91.2, 92.2)	93.3 (92.6, 93.9)	90.1 (89.4, 90.9)	Reference
One	743		6.6 (6.2, 7.1)	5.5 (4.9, 6.0)	7.8 (7.1, 8.5)	1.4 (1.2, 1.6)
At least 2	183		1.6 (1.4, 1.9)	1.3 (1.0, 1.6)	2.0 (1.7, 2.4)	1.5 (1.2, 2.1)

Abbreviations: D.K., I do not know; *IQR* Interquartile range; *OR* Odds ratio

^a^ Q1 = ”*Was it a glomerulonephritis?*”; Q2 = “*Was it a pyelonephritis?*”; Q4 = ” Was it a hereditary or congenital kidney disease (including polycystic kidney disease)?”; Q5 = ”*Have you ever been told that you have kidney stones?*””; Q6 = ”*Have you ever been told that you have another kidney disease, not mentioned yet?*”; Q7 = ”*Has a doctor ever told you that you have a reduced kidney function or a renal failure?*”; Q12 = ”*Have you undergone a renal surgery for another reason?*”

^b^Obtained with the Clopper-Pearson method. Participants reporting ‘I do not know’ to the respective question were excluded from this analysis

^c^ Estimated using age-adjusted logistic linear models. Participants reporting ‘I do not know’ to the respective question were excluded from this analysis

^d^Obtained with the Wald method

^e^Estimated using age-adjusted logistic regression models, taking ‘None’ as reference category

Distributions of the time from diagnosis and sex-associated risks of reporting are also included

**Fig. 1:**
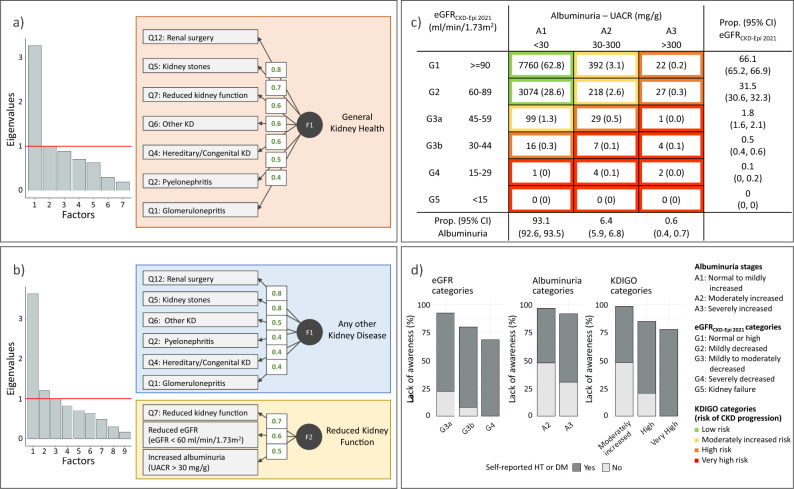
**Panel A**: Factor analysis results. Scree plot and graphical summary with factor loadings based on the questionnaire only. **Panel B**: Factor analysis results. Scree plot and graphical summary with factor loadings based on both the questionnaire and the marker-based diagnoses. **Panel C**: KDIGO CKD stages calibrated to the general population distribution, with multinomial 95% confidence intervals. **Panel D**: Awareness of CKD across CKD severity stages

All questionnaire items displayed low sensitivity and high specificity in identifying reduced eGFR or increased albuminuria (Supplemental Table S3). For instance, reporting a diagnosis of any kidney disease (Q0) showed sensitivity and specificity of 0.27 and 0.92 for reduced eGFR, and 0.13 and 0.92 for increased albuminuria, respectively. Q7 (“*Has a doctor ever told you that you have a reduced kidney function or a renal failure?*”) exhibited a sensitivity of 0.08 and a specificity of 0.99 for reduced eGFR, and 0.02 and 0.99 for increased albuminuria. Factor analysis of the questionnaire items identified one single meaningful factor named ‘general kidney health status’, to which many items contributed substantially equally (Fig. 1a). When including increased albuminuria and reduced eGFR in the factor analysis, two distinct factors emerged: one was defined as ‘reduced kidney function’, represented by the proximity between increased albuminuria and reduced eGFR with the question “*Has a doctor ever told you that you have a reduced kidney function or a renal failure?*” (Q7); the second was defined as ‘any other kidney disease’ and corresponded to the combination of all other items except Q7 (Fig. 1b).

Participants responding ‘I do not know’ to Q7 (“*Has a doctor ever told you that you have a reduced kidney function or a renal failure?*”), exhibited eGFR and UACR distributions similar to those responding ‘Yes’ and significantly different from those responding ‘No’ (Supplemental Figure S6a-b): compared to those responding ‘No’, those responding ‘I do not know’ had lower mean eGFR of −10.6 ml/min/1.73m^2^ (95%CI: −14.3, −6.9) and higher mean logUACR of 0.1 log(mg/g) (95%CI: −0.1, 0.4), and those responding ‘Yes’ had lower mean eGFR of −11.7 (95%CI: −14.2, −9.3) ml/min/1.73m^2^ and higher mean logUACR of 0.5 (95%CI: 0.3, 0.7) log(mg/g). Regression models on eGFR and logUACR against combinations of hypertension, diabetes mellitus, and Q7 responses, showed that, in the presence of hypertension or diabetes mellitus, those responding ‘I do not know’ had a profile more compatible with the presence rather than absence of CKD; in contrast, in the absence of hypertension and diabetes mellitus, their values were distributed like those of individuals without CKD (Supplemental Figure S7a-b).

### CKD prevalence and awareness

CKD prevalence varied depending on the definition (Table 3). The population-representative estimate of kidney dysfunction was 8.59% (95%CI 8.09–9.12%), driven by increased albuminuria (prevalence = 6.95%, 95%CI 6.49–7.42%) rather than reduced eGFR (prevalence = 2.42%; 95%CI 2.15–2.72%). The KDIGO classification (Fig. 1c) identified no participants in eGFR stage G5 and a 0.6% prevalence (95%CI 0.4–0.7%) of albuminuria stage A3. Overall, 0.3% (95%CI 0.2–0.4%) of individuals had a very high risk of CKD progression, incident CVD events and mortality.

**Table 3:** Prevalence of kidney diseases in the Val Venosta/Vinschgau district using different definitions

Definition	Prevalence (95% confidence interval)			OR (F vs M)
	Overall	Males	Females	
(1) CKD_SR_: Questionnaire self-rep. reduced kidney function (Q7^a^)	0.69 (0.55, 0.86)	0.64 (0.45, 0.88)	0.74 (0.54, 1.00)	1.10 (0.71, 1.71)
(2) Reduced eGFR (eGFR < 60 ml/min/1.73 m^2^)	2.42 (2.15, 2.72)	1.66 (1.35, 2.03)	3.19 (2.75, 3.67)	1.72 (1.31, 2.25)
(3) Increased albuminuria (UACR > 30 mg/g)	6.95 (6.49, 7.42)	5.62 (5.05, 6.24)	8.28 (7.58, 9.02)	1.44 (1.24, 1.67)
(4) Kidney dysfunction—(2) or (3)	8.59 (8.09, 9.12)	6.77 (6.14, 7.44)	10.43 (9.66, 11.25)	1.51 (1.32, 1.73)
(a) Possible CKD: CKD_SR_ or kidney dysfunction	9.05 (8.53, 9.58)	7.17 (6.52, 7.86)	10.94 (10.14, 11.77)	1.50 (1.31, 1.71)
(b) Kidney disease of any kind	15.76 (15.1, 16.43)	12.54 (11.7, 13.41)	19.00 (18.00, 20.03)	1.58 (1.42, 1.75)

OR, Odds Ratio; CKD, chronic kidney disease

^a^Q7: “*Has a doctor ever told you that you have a reduced kidney function or a renal failure?*”

In contrast, CKD_SR_ prevalence was low: 0.69% (95%CI 0.55–0.86%). Prevalence of possible CKD (kidney dysfunction or CKD_SR_) was 9.05% (95%CI 8.53–9.58%). Except for CKD_SR_, prevalence was always higher in females than males. Overall, 15.76% (95%CI 15.10–16.43%) of the district’s adult population had experienced at least one kidney disease of any kind.

Of the 822 individuals with kidney dysfunction, only 31 reported CKD_SR_. Thus, the standardized proportion of individuals who were not aware of having a kidney dysfunction was 95.3% (95%CI 93.8–96.5%). The lack of awareness decreased with increasing disease severity (Fig. 1d; Supplemental Table S4), albeit heterogeneously across outcomes: lack of awareness was > 90% among albuminuria stage A3 individuals and 79.6% among those with eGFR stage G4 (Supplemental Table S4). Most unaware individuals reported having hypertension or diabetes mellitus. This occurred in approximately two-thirds of the individuals with albuminuria stage A3 and 100% of those with eGFR stage G4 (Fig. 1d).

## Discussion

Our investigation of CKD and kidney health in the Val Venosta/Vinschgau district (South Tyrol, Italy), combining self-reported data and biomarkers, reveals a prevalence of possible CKD of approximately 9%. It offers firsthand experience with implementing a dedicated kidney health questionnaire outside clinical settings and quantifies lack of CKD awareness in the general population.

Although a single eGFR and UACR assessment may overestimate the presence of CKD,[24] the observed prevalence of possible CKD was lower than the 12.7% to 15.5% reported by some Italian population cohort studies (INCIPE [4] and SardiNIA [25], respectively), yet higher than others [5, 6], while generally matching figures from other western European countries [3]. Sharing geographic and cultural proximity with the CHRIS study, the KORA study reported prevalence of reduced eGFR and kidney dysfunction of 9.7% and 16.0%, respectively [26].

To our knowledge, no prior study has systematically investigated the prevalence of a broad spectrum of self-reported kidney diseases in the general population. To address this gap, we implemented the novel CHRIS kidney questionnaire. Participants reporting at least one kidney disease exceeded 7%. The most reported condition was pyelonephritis (3%), with an almost ninefold higher likelihood of reporting for females than males, consistent with literature indicating a higher risk of urinary tract infections, including pyelonephritis, in females [27]. In addition to clinical reasons, the higher self-reported prevalence of both pyelonephritis and glomerulonephritis in females could also reflect under-reporting among males [28]. Among the other conditions, congenital diseases were reported by 0.2% and kidney stones by nearly 3% of the sample.

All conditions presented a high-to-nearly-perfect specificity and low-to-null sensitivity regarding kidney dysfunction, reflecting that the listed diseases do not necessarily affect eGFR and UACR, except in the long term. Questions on dialysis, transplantation, and renal surgeries yielded no insight, with almost no participants reporting such conditions. This suggests caution when selecting survey questions for the general population, as their inclusion may cause substantial difficulties for both the interviewer and the interviewee, with minimal or no benefit, potentially compromising response accuracy.

The questionnaire was able to effectively identify kidney diseases with a specific diagnosis and symptoms identifiable by both patients and physicians from CKD, which often progresses latently and asymptomatically even when diagnosed. This finding is reflected by factor analysis results including both CKD_SR_ and measured kidney function markers: CKD_SR_ clustered well with measured eGFR and UACR levels, while all other questionnaire items clustered apart. This observation supports the importance of implementing kidney health questionnaires for population studies as they capture disease domains not typically identified by routinely measured markers.

A substantial majority of individuals with kidney dysfunction was unaware of having this condition, aligning with recent literature reporting underdiagnosis rates ranging from 61.6% in the USA to 95.5% in France [9]. Our results could reflect a lack of renal health education among participants, stemming from limited attention to CKD by health providers. It is noteworthy that CKD was included in the new essential assistance levels, a set of essential healthcare services and standards established by the Italian government in November 2017 [29]. Therefore, many healthcare authorities might not yet have implemented measures to raise attention toward CKD during the 2011–2018 data collection period. As the results show, awareness was poor even in the presence of diagnosed diabetes or hypertension. This could imply suboptimal compliance with guidelines recommending periodic creatinine assessments for hypertensive [30] or diabetic [31] individuals. Alternatively, individuals diagnosed with diabetes or hypertension might not receive a formal CKD diagnosis due to reduced kidney function being assumed as a standard comorbidity. Given kidney dysfunction was more common in females, while CKD_SR_ prevalence was similar between sexes, under-reporting or lack of awareness is higher in women than men, consistent with literature indicating a lower likelihood of CKD diagnosis, monitoring, and management in women [32, 33]. Consequently, while some underdiagnosis is generally expected for a silent disease like CKD, even in electronic health records [7], addressing CKD underdiagnosis becomes imperative also as a way to reduce gender inequalities in healthcare provision.

Strengths of our analyses are the large sample size, calibration to the target population to obtain representative estimates and mitigate selection bias, and the simultaneous availability of serum creatinine and UACR, which allowed to better gauge the KDIGO criteria.

There are also several limitations. Due to logistic and motivational issues, one of the drawbacks of population-based studies like CHRIS lies in the difficulty of recruiting chronically ill elderly individuals. Indeed, the most severe CKD stages are not represented in the CHRIS study: this may have caused some underestimation of our figures, despite our attempt to overcome the problem via calibration to the population distribution. In addition, despite the revisions made to the questionnaire, we cannot override poor question wording, potentially leading to underestimation and perhaps even misclassification. Furthermore, we cannot exclude recall bias due to confusion or forgetfulness by the patient, not least because some diagnoses dated significantly back in time. Moreover, questions required the use of technical terminology, which may have been unfamiliar to the participants, also because clinicians typically communicate with patients by explaining symptoms and consequences rather than using medical jargon, difficult for the average patient to comprehend. Given the broad scope of the CHRIS study, it was not feasible to employ field-specific study assistants. Study assistants were trained to strictly adhere to the questionnaire wording to limit operator bias and maintain consistency in data collection. This might have caused some classification bias by participants not recognizing the specific terminology being used in the questions. On the other hand, terms like glomerulonephritis, for instance, are just generic, as they include various specific subpathologies, like IgA nephropathy, lupus nephritis or membranous nephropathy.[11] However, including these specific terms might not be worthwhile as these are rarer conditions and may represent an unfamiliar vocabulary for study participants. Therefore, it seems reasonable to assume that the estimated prevalence of pyelonephritis and glomerulonephritis is an underestimation of the real presence in the reference population. Electronic health record linkage might overcome these limitations, however this was not yet implemented in the CHRIS study. Our questionnaire addresses kidney diseases in general, most of which are likely chronic and would typically fall under the CKD definition. One exception is stone disease, a highly prevalent urinary disorder, which is not generally included among CKD conditions. For this reason, we report separate estimates of the prevalence of any kidney disease and of possible CKD.

Other limitations pertain to the measured markers: neither measured GFR nor cystatin C, that would have provided more reliable GFR estimates, was available. However, assessing eGFR through the creatinine-based CKD-EPI equation allowed comparison to most available population-based studies. Nevertheless, according to KDIGO guidelines, a single abnormal determination of GFR and/or UACR does not establish the chronicity required for a CKD diagnosis, limiting our ability to definitively diagnose CKD in this study. Despite these limitations, and although we cannot infer CKD prevalence from our data on “kidney disease of any kind”, our estimates on kidney dysfunction, reduced eGFR and increased albuminuria can still be meaningfully compared to those obtained by the majority of studies on CKD prevalence in different populations worldwide, which also relied on a single measurement[2]. Finally, although the occurrence of acute events could theoretically influence our findings, the relatively stable clinical condition of study participants likely mitigates the impact on the prevalence estimates.

In conclusion, understanding the coverage of CKD diagnosis and the extent of the individuals’ awareness about their CKD status and the associated risk factors is crucial in promoting early diagnosis and effective management. Enhancing awareness can empower individuals to adopt healthier lifestyles and engage in proactive healthcare behaviors, thus reducing CKD burden on individuals and healthcare systems. While producing population-representative estimates of kidney disease prevalence in the study area, the CHRIS study provides a rare example of a kidney health questionnaire for general population studies. The extreme lack of awareness toward kidney dysfunction reflects the international context, reinforcing the need for more accurate tools to assess kidney diseases in the general population.

## Supplementary Information

Below is the link to the electronic supplementary material.

Supplementary file1 (DOCX 1410 KB)

## Data Availability

CHRIS study data and samples can be requested for research purposes by submitting a dedicated request to the Eurac Research Institute for Biomedicine Access Committee at access.request.biomedicine@eurac.edu.
